# 502 Long-term Renal Recovery in Burn-related Acute Kidney Injury Requiring Continuous Renal Replacement Therapy

**DOI:** 10.1093/jbcr/irae036.137

**Published:** 2024-04-17

**Authors:** Travis D Gordon, Anthony Papp, Bader Al-Zeer

**Affiliations:** University of British Columbia, Vancouver, BC; Vancouver General Hospital, Vancouver, BC; University of British Columbia, Vancouver, BC; Vancouver General Hospital, Vancouver, BC; University of British Columbia, Vancouver, BC; Vancouver General Hospital, Vancouver, BC

## Abstract

**Introduction:**

Acute Kidney injury (AKI) is a common complication of severe burn injury associated with significant morbidity and mortality. Continuous Renal Replacement Therapy (CRRT) is the modality of choice int he treatment of burn-related AKI. The purpose of this study was to look at long-term renal function of burn survivors who required CRRT during their acute stay in ICU as a result of burn-related AKI.

The hypothesis of this study is burn patients who develop burn-related AKI requiring supplemental CRRT during their hospitalization, have return or normal/baseline renal function long-term.

**Methods:**

A retrospective cohort study looking at burn patients at a single institution. Inclusion criteria includes: Adult (> 18yo) burn patients with KDIGO stage 1-3 AKI subsequent to burn injury who received CRRT during ICU admission. Exclusion criteria included preceding chronic kidney disease, kidney transplant. Retrospective data was collected between January 2012 - December 2021 using the institutions Burn registry, and ICU and Renal databases. Renal follow-up was collected up to 3 years post discharge. Statistical analyses was done using Microsoft Excel including T-tests, and multivariate analyses.

**Results:**

Of the 31 patients who met inclusion criteria, 22 survived their burn injuries, a mortality rate of 31.8%. There were no significant differences in demographics, comorbidities, burn characteristics, or critical care interventions between patients who survived vs. those who perished. Serum creatinine values and eGFR values on average normalized to patient baseline at discharge and remained stable through follow up for 91% of patients. 4/22 patients required dialysis after their discharge from hospital (18.2%), 3 within the first year of discharge, but none required dialysis > 3 years after discharge. 5 patients died within 3 years from discharge, 4 of whom died within the first year.

**Conclusions:**

AKI in the setting of severe burn injury is common, often necessitating the need for CRRT. Despite high rates of conversion to chronic kidney disease in the General ICU cohort who develop AKI necessitating CRRT, burn-related AKI requiring CRRT results in much lower rates of conversion to ongoing renal dysfunction. Our cohort showed a return of baseline renal function in 20/22 patients who survived their burn injury (91%) in long-term (> 3years) follow-up. Mortality rates in our cohort were also lower than previously cited literature.

**Applicability of Research to Practice:**

Within our cohort, 91% of our patients had return to baseline renal function after their burn-related AKI, and a mortality rate of 31.8% which is lower than previously cited studies. Given all survivors received CRRT, this could highlight the potential benefit CRRT offers to burn patients in the long-term. Secondly we reported unsatisfactory low rates of long-term renal function follow-up data for burn related AKI, thus highlighting room for improvements in practice.

